# The Catestatin-Derived Peptides Are New Actors to Fight the Development of Oral Candidosis

**DOI:** 10.3390/ijms23042066

**Published:** 2022-02-13

**Authors:** Davide Mancino, Naji Kharouf, Francesco Scavello, Sophie Hellé, Fouad Salloum-Yared, Angela Mutschler, Eric Mathieu, Philippe Lavalle, Marie-Hélène Metz-Boutigue, Youssef Haïkel

**Affiliations:** 1 Department of Biomaterials and Bioengineering, INSERM UMR_S 1121, University of Strasbourg, 67000 Strasbourg, France; mancino@unistra.fr (D.M.); francescoscav@tiscali.it (F.S.); s.helle@unistra.fr (S.H.); angela.mutschler@u-bordeaux.fr (A.M.); eric.mathieu@inserm.fr (E.M.); philippe.lavalle@inserm.fr (P.L.); marie-helene.metz@inserm.fr (M.-H.M.-B.); youssef.haikel@unistra.fr (Y.H.); 2 Department of Endodontics and Conservative Dentistry, Faculty of Dental Medicine, University of Strasbourg, 67000 Strasbourg, France; 3 Pôle de Médecine et Chirurgie Bucco-Dentaire, Hôpital Civil, Hôpitaux Universitaire de Strasbourg, University of Strasbourg, 67000 Strasbourg, France; 4 Department of Medical Laboratory, The General Authority of the Syrian Arab Red Crescent Hospital, Damascus 0100, Syria; fouad.yar@gmail.com

**Keywords:** candidosis, resistance, antifungal, antimicrobial peptides, Catestatin

## Abstract

Resistance to antifungal therapy of *Candida albicans* and non-*albicans Candida* strains, frequently associated with oral candidosis, is on the rise. In this context, host-defense peptides have emerged as new promising candidates to overcome antifungal resistance. Thus, the aim of this study was to assess the effectiveness against *Candida* species of different Catestatin-derived peptides, as well as the combined effect with serum albumin. Among Catestatin-derived peptides, the most active against sensitive and resistant strains of *C*. *albicans*, *C. tropicalis* and *C. glabrata* was the *D*-isomer of Cateslytin (*D*-bCtl) whereas the efficiency of the *L*-isomer (*L*-bCtl) significantly decreases against *C. glabrata* strains. Images obtained by transmission electron microscopy clearly demonstrated fungal membrane lysis and the leakage of the intracellular material induced by the *L*-bCtl and *D*-bCtl peptides. The possible synergistic effect of albumin on Catestatin-derived peptides activity was investigated too. Our finding showed that bovine serum albumin (BSA) when combined with the *L*- isomer of Catestatin (*L*-bCts) had a synergistic effect against *Candida albicans* especially at low concentrations of BSA; however, no synergistic effect was detected when BSA interacted with *L*-bCtl, suggesting the importance of the C-terminal end of *L*-bCts (GPGLQL) for the interaction with BSA. In this context in vitro *D*-bCtl, as well as the combination of BSA with *L*-bCts are potential candidates for the development of new antifungal drugs for the treatment of oral candidosis due to *Candida* and non-*Candida albicans*, without detrimental side effects.

## 1. Introduction

*Candida* species are part of the commensal flora of the mucous membranes and are opportunistic pathogens. Under appropriate conditions, they can be responsible for oral candidosis, the most common oral yeast mycoses encountered in humans [1]. *Candida albicans* is the most frequently isolated species associated with oral candidosis, accounting for between 60% and 75% [2,3] of cases and involving between 41% and 30% of candidemia episodes. However, over the last few years, non-*albicans Candida* strains, responsible for oral candidosis, have tended to increase, and together they cause the majority of *Candida* bloodstream infections, even though with important differences across countries and continents [4,5,6,7]. Recent epidemiological studies have reported an increased incidence of non-*albicans* candidemia, especially in the USA, Northern Europe, and Australia, due to *Candida glabrata* and *Candida tropicalis*, which are less susceptible to antifungal drugs and could be associated with high morbidity and high mortality [8,9,10]. These epidemiological data pose a growing threat to public health worldwide and the Interagency Coordination Group on antimicrobial resistance (IACG) has already expressed concerns about the increase in resistant pathogens, which could lead to overall treatment inefficiency [11]. In this context, there is a pressing demand for new active anti-*Candida* drugs.

Host-defense peptides (HDPs) have emerged as new attractive candidates in the development of new antimicrobial agents [12]. In addition, HDPs have been proven to be potent immune effectors via the orchestration of immune responses [13]. Furthermore, it has been amply demonstrated that the pro-hormone chromogranin A (CgA) is the precursor of several physiological HDPs [14,15,16,17] released under stress in most body fluids and saliva [18]. One of these HDPs, Catestatin (bCts, bovine CgA 344–364), initially described as a potent inhibitor of catecholamines release [19], displays direct antimicrobial activities [17], contributes to immune regulation, and may modulate severe inflammatory response [20,21,22,23]. It has been established that Cateslytin (bCtl, bovine CgA344358) is the active core of bCts [17] and that it resists bacterial degradation [24]. bCts and bCtl show a broad spectrum of antimicrobial activities against bacteria and yeasts when attached to biomaterials [25,26]. Additionally, we have previously characterized the antimicrobial and mechanistic properties of the *D*-isomer of bCtl where all *L*-amino acids have been replaced by *D*-amino acids [27,28,29].

A crucial role could be played by albumin, an indispensable molecule in the transport, distribution, and metabolism of many endogenous and exogenous ligands including small peptides and numerous pharmaceutical products. A recent study even reported that albumin could modulate *C*. *albicans* pathogenicity [30], whereas further work showed that continuous infusion of 4% albumin was effective in reducing care-related infections in shock patients by increasing the availability of HDP Vasostatin-I [31].

The aims of the present study are multifaceted: firstly, to carry out a drug-design study to characterize the most potent antimicrobial peptide derived from bovine and human Cts (bCts and hCts) able to inhibit the growth of different *Candida* strains, secondly to examine *L*- and *D*-bCtl-*Candida* interaction, thirdly to investigate the activity against *C. albicans* of *L*-bCts/*L*-bCtl combined with albumin and finally to characterize the interaction of these peptides with albumin by using biophysical techniques.

## 2. Results

### 2.1. Characterization of the Most Active Peptide against Candida albicans

Different sensitive “S” and resistant “R” *Candida* strains characterized as shown in Table 1 were used. For a first study, differently designed peptides derived from bovine Catestatin (bCgA344–364) and human Catestatin (hCgA 352–372) (Table 2a) were tested against *C. albicans* (“S” and “R”). The MIC (µg/mL) is reported for each test (Table 2). Since, in vivo, N- and C- terminal modifications provide simple but useful approaches to improve peptide stability and effectiveness [32], we designed several peptides with shorter sequences than b/hCts including N-terminal modification (Ac, acetylation, and Pa, Palmatoylation) and C-terminal addition of tryptophane residue (W) (Table 2). Most amino acids (except Glycine), have 1 or 2 asymmetric atoms and can thus form enantiomers (*L*- and *D*-). The *D*-peptide can reduce susceptibility to proteolysis but also shows antimicrobial activity because the helical structure and amphiphilicity are unchanged.

Among the bovine sequences (Table 2), the most active peptide corresponds to the *D*-form of bCtl with a MIC of 5.5 µg/mL and 9.6 µg/mL against the “S” and “R” forms of *C. albicans,* respectively, whereas among the *L*-hCtl the most active peptide is the complete *L*-hCtl sequence with a MIC of 50 µg/mL for the “S” and “R” forms of *C. albicans.* These data demonstrate the important role of the Arginine (R) residue at the N-terminal position in the bCtl sequence and of the *D*-structure as previously demonstrated for other microorganisms [27,28,29].

### 2.2. Antimicrobial Activity of L- and D-bCtl against C. tropicalis and C. glabrata

The antimicrobial activity of *L*- and *D*-Ctl are similar against *C. albicans* and *C. tropicalis* (“S” and “R”) and the present data confirm the highest activity of *D*-bCtl against *C. glabrata* (“S” and “R”). It is also important to highlight the low MIC (2 µg/mL) obtained for *L-* and *D*-Ctl against *C. tropicalis* “R” (Table 3).

### 2.3. TEM Analysis of L- and D-bCtl against “S” and “R” C. albicans

**Figure 1:**
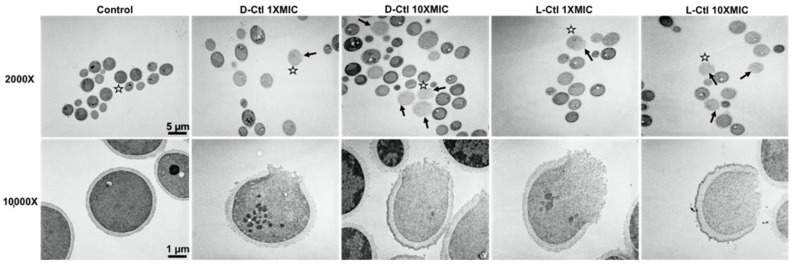
Transmission electron microscopy images of cells from “S” *C. albicans,* treated with *D*-bCtl or *L*-bCtl at different concentrations, compared to untreated cells. *D*-bCtl or *L*-bCtl were used at concentrations of 1 × MIC and 10 × MIC and incubated for 1 h at 37 °C with minimal shaking. White stars indicate which cell is magnified at 10,000×. Black arrows localize lysed cells.

**Figure 2:**
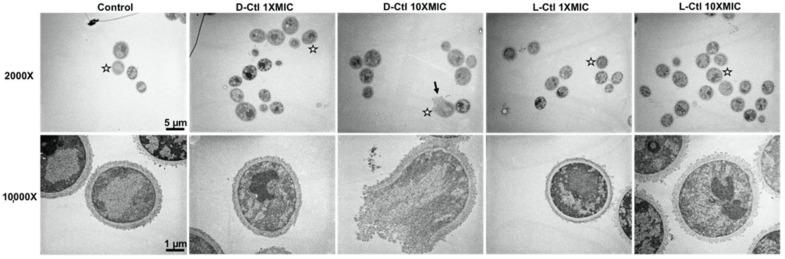
Transmission electron microscopy images of cells from a resistant strain of *Candida albicans,* treated with *D*-Ctl or *L*-Ctl at different concentrations, compared to untreated cells. *D*-Ctl or *L*-Ctl were used at concentrations of 1 × MIC and 10 × MIC and incubated for 1 h at 37 °C with minimal shaking. White stars indicate which cell is magnified at 10,000×. Black arrows localize lysed cells.

The interaction of the peptides with *C. albicans* was examined by TEM in order to investigate the mechanism used by *L*- and *D*-Ctl to inhibit the *C. albicans* growth. This technique shows the permeabilization of the fungal membrane and the variable efficiency of *L*-bCtl and *D*-bCtl towards “S” and “R” *C. albicans* (Figure 1 and Figure 2). TEM images clearly demonstrate fungal membrane lysis and secretion of intracellular material induced by both peptides (Figure 1 and Figure 2). These images show a higher proportion of cell lysis of the sensitive strain of *C. albicans* when the concentrations of *L*-Ctl or *D*-Ctl increase (Figure 1), whereas the proportion of cell lysis appears lower for “R” *C. albicans* (Figure 2).

### 2.4. Antimicrobial Activity of L-bCtl Combined with Albumin against C. albicans

**Figure 3:**
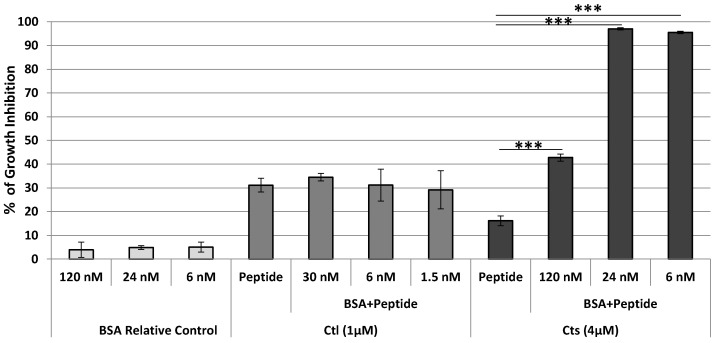
Antimicrobial activity of *L*-bCtl and *L*-bCts with BSA against *C. albicans*. BSA was used with a molar concentration of 30, 6, and 1.5 nM for Ctl (1 µM) or 120, 24 and 6 nM for *L*-bCts (4 µM). The growth inhibition of BSA (controls) is reported only for the concentrations used with *L*-bCts where we obtained synergistic antimicrobial effects. No antimicrobial effects were obtained for BSA combined with *L*-bCtl., *** *p* ≤ 0.001 with 1 way ANOVA comparing peptide only to BSA plus peptide treatments.

BSA was not active at the molecular concentrations tested. Since the MIC ratio (L-bCts)/MIC (L-bCtl) is close to 4 (Table 2), 1 µM *L*-bCtl and 4 µM *L*-bCts were combined with BSA (1.5, 6, 30 nM) and (6, 24, 120 nM) respectively and tested against *C. albicans*. BSA was unable to improve the antimicrobial activity of 1µM *L*-bCtl with a growth inhibition ratio evaluated at 30%. In contrast, with 4 µM *L*-bCts and the same BSA/peptide, ratio, more than 90% of inhibition was achieved with 24 nM BSA (Figure 3). These data show that the combination *L*-bCts and BSA have a synergistic effect against *C. albicans* as well as that the C-terminal sequence of *L*-bCts (GPGLQL) is important for the interaction with BSA. The synergistic effect between BSA and *L*-bCtl is obtained especially when BSA concentrations are low (6 and 24 nM), in contrast to high BSA concentrations of 120 nM where the antimicrobial activity against *Candida* was not improved (see discussion part).

**Figure 4:**
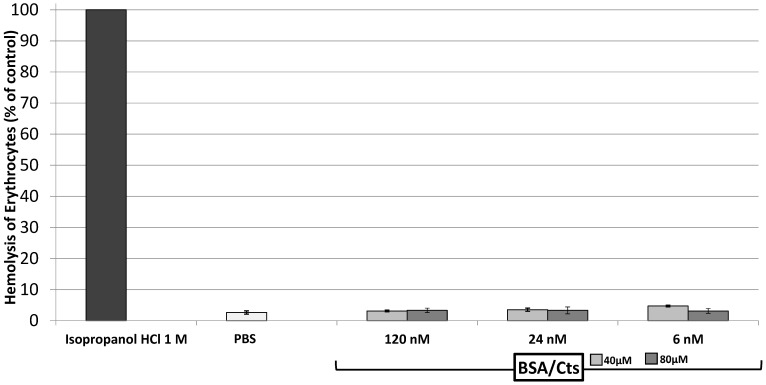
Non-toxicity of BSA/*L*-bCts complex. Hemolytic assays of erythrocytes with BSA (120, 24, and 6 nM) plus *L*-bCts (40 and 80 µM). All the treatments were statistically significant compared to the positive control of the hemolytic test with One way ANOVA.

We have also demonstrated the non-toxicity of the combination *L*-bCts with BSA (Figure 4).

### 2.5. Biophysical Study of the Interaction Cts-Albumin

In order to understand the synergistic effect of albumin on *L*-bCts activity, we investigated a possible interaction between *L*-bCts/*L*-bCtl and BSA using QCM.

**Figure 5:**
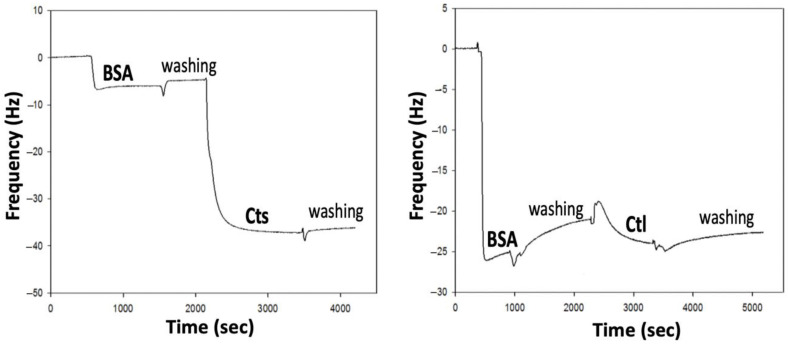
Quartz Crystal Microbalance (QCM) analysis of the interaction BSA with *L*-bCts or *L*-bCtl. The analysis was performed at 37 °C in PBS pH 7.2 with a first injection of 2 mg/mL BSA (400 µL,) and a second injection of 1 mg/mL *L*-bCts or *L*-bCtl (400 µL).

BSA was first deposited on the top of the quartz crystal and a change in the third frequency resonance was measured, corresponding to the variation of $\frac{∆f_{\nu }}{\nu }=$ 7 Hz (with ν = 3). The next deposition was performed with Cts with a frequency variation of $\frac{∆f_{\nu }}{\nu }=$ 22 Hz (Figure 5). According to Sauerbrey’s approximation, the deposited mass was calculated following the relation:

$$m=-C\frac{∆f}{\nu }$$

where *C* is a constant characteristic of the crystal plate (*C* ≈ 17.7 ng·cm^−2^·Hz^−1^ for the quartz plates used here).

In our case, the deposited masses of BSA and *L*-bCts were 88.5 and 566.4 ng·cm^−2^ respectively, corresponding to a massic ratio *L*-bCts/BSA of 6.4 or a molar ratio of 177. These data indicate the presence of significant interactions between *L*-bCts and BSA.

On the opposite, under the same experimental conditions, a second deposition with *L*-bCtl instead of *L*-bCts was performed but the results indicate almost no frequency variation, corresponding to an absence of interaction between BSA and *L*-bCtl (Figure 5).

QCM analysis indicates that *L*-bCts interacts with BSA but not *L*-bCtl (Figure 5), suggesting the importance of the C-terminal end of *L*-bCts (GPGLQL) (see Table 2). These data confirm the antimicrobial activities previously obtained for BSA-*L*-bCts/*L*-bCtl combinations (Figure 3).

## 3. Discussion

Previous epidemiological studies have confirmed that fungal infections will certainly be a growing concern in the years to come, as resistance of *C. albicans* as well as non-*albicans Candida* strains to existing antifungal drugs is becoming more and more prevalent [8,9,10]. In this context, the development of new antifungal drugs remains a major challenge to overcome the prevalence of drug-resistant fungal pathogens. Over the last few years, an increasing interest in the development of new antifungal therapeutic agents based on the pharmaceutical exploitation of HDPs is spreading [12,25,26]. The present study showed that *L*- and *D*-bCtl, as well as the combination of *L*-bCts with BSA exhibit an antimicrobial activity against *Candida* strains. With regard to *C. albicans* “S” *L*-bCtl, *D*-bCtl and *L*-bCts combined with 24 nM BSA are active with a MIC of 7.9 µM, 5.5 µM and 4 µM, respectively.

Our previous paper [28] demonstrated the interesting activity of P1, (*D*-bCtl) against *C. albicans*. The others P2-P11 peptides were designed to evaluate the activity of shorter fragments and to define the structural domain for the activity. These peptides were designed taking into account the role of the N-terminal sequences (344–351; RSMRLSFR) and (348–358; LSFRARGYGFR) for the antimicrobial activities [17]. The role of the LSFR tetrapeptide was emphasized by our previous study showing that it participates in the inhibition of microbial enzymes [33]. Of great interest, the N- and C-terminal modifications (Ac, Pa, and W) added in P3-P7, P10-P11 sequences are unable to improve the anti-*Candida* activities and the helical domain (S6 to Y12) of *L*- and *D*-bCtl is important for the anti-*Candida* activity [34].

Our results based on antifungal assays demonstrated that *D*-bCtl is a potent antifungal agent against sensitive and resistant strains of *C. albicans*, *C. tropicalis,* and *C. glabrata* (Table 2 and Table 3), whereas the efficiency of *L*-bCtl significantly decreases against *C. glabrata* strains, as the MIC values obtained in the presence of the resistant strain o *C. glabrata* strain increased to 61.4 µg/mL for *L*-bCtl and remained stable for *D*-bCtl, with a value of 15.0 µg/mL.

The present study demonstrated the improved efficiency of *D*-bCtl compared to *L*-bCtl against a wide range of *Candida* strains. This could be due to the high stability of bCtl [24] and more particularly the *D*-isomer [27,28].

Images obtained by transmission electron microscopy (TEM) demonstrated the permeabilization of the fungal membrane and the leakage of the intracellular material induced by the *L*-bCtl and *D*-bCtl peptides after a short incubation time (1 h) in the presence of the pathogen (Figure 2 and Figure 3). We have previously reported that electrostatic interactions between the arginine residues of the positively charged *L*-bCtl peptide sequence and the negatively charged membrane lipids are responsible for the formation of pores of 1 nm diameter across the membrane [27,28]. Indeed, the thicker and stiffer membrane domains that result from these interactions generate membrane defects that allow peptide passage across membranes and ultimately lead to membrane breakage. A previous study demonstrated the role of ergosterol in facilitating the penetration of a CgA-derived peptide into membranes [35]. Furthermore, at the intracellular level, we showed that *L*-bCts are able to interact with Calmodulin to inhibit intracellular calmodulin-binding enzymes such as Calcineurin [21]. In this context, the bCts-derived peptides represent innovative and potent HDPs.

No less important is the role that albumin may play in enhancing the activities of *L*-bCts. This finding is different from that reported for others HDPs [36,37]. Our hypothesis is that the Cts-BSA combination may protect the peptide from proteolytic degradation and improve its biodisponibility, as reported for several peptides [38,39]. In addition, these data have been supported by our clinical study in collaboration with the Intensive Care Unit of Strasbourg showing that infusion with a low concentration of albumin improves the recovery of patients with nosocomial infections. Compared to 20% albumin infusion, we showed that continuous 4% albumin is effective in reducing care-related infections in shock patients by increasing the availability of antimicrobial peptides. Due to its anti-oxidative properties, albumin prevents peptide oxidation. In the bCtl sequence, several residues (M, F, and Y) may be oxidized and induce structural modifications. The combination with albumin may prevent the oxidation of these residues.

Finally, preventive antifungal protection of the oral mucosa is a long-term research prospect. This preventive approach could be based on surface modifications of medical devices such as removable dental prothesis, which are partly responsible for the emergence of oral candidosis. As such, the use of a surface coating functionalized with *D*-bCtl might help reduce infections associated with medical devices. Nevertheless, research for the development of preventive or therapeutic means of action against oral candidosis should be continued. This study will be extended by the analysis of the interaction of bCtl on the other *Candida* strains. In vivo studies will be performed with infected animal models in order to compare the effects of bCtl (*L* or *D*- forms) and the combination BSA and *L-* or *D*-bCts.

## 4. Materials and Methods

### 4.1. Preparation and Characterization of Synthetic Cts and Its Derived-Peptides

Chemically synthesized peptides derived from bovine-Catestatin (bCts) and human-Catestatin (hCts) were supplied by Pepmic (Suzhou, China). The purity of these peptides was tested by reverse phase (RP) HPLC with a Dionex HPLC system (Ultimate 3000; 13 Sunnyvale, CA, USA) on a Vydac 208 TP C8 column (2.1 × 150 mm) equipped with a Vydac 208 TP 14 pre-column (7.5 × 2.1 mm) (Vydac, AIT France, Houilles, France) according to the previously described method [24].

### 4.2. Antimicrobial Assays against the Different Strains of Candida

Strains of *Candida albicans*, *Candida tropicalis* and *Candida glabrata*, frequently found in cases of oral candidosis, were tested for *L*-bCtl and *D*-bCtl antimicrobial activity. The sensitive strain of *C. albicans* (ATCC 10231^TM^) was provided by ATCC (Manassas, VA, USA). The resistant strain of *C. albicans*, the sensitive and resistant strains of *C. tropicalis* as well as the sensitive and resistant strains of *C. glabrata* were kindly provided by Dr Valérie Letscher-Bru (Laboratory of Parasitolology and Medical Mycology, Strasbourg, France). The susceptibility of each strain is reported in Table 1.

Each *Candida* strain was first plated on agar plates and cultured for 24 h at 37 °C. After incubation, one colony per isolate was transferred into 5 mL of Sabouraud medium pH 5.6 (Sigma-Aldrich, Le Pont-de-Claix, France) supplemented with tetracycline (10 μg/mL) and cefotaxime (10 μg/mL) and incubated aerobically under agitation at 37 °C for 24 h. The resulting cultures were resuspended at an absorbance of 0.001 (OD_600nm_ = 0.001) in Sabouraud medium. The OD_600nm_ was evaluated with a spectrophotometer (BIO-RAD Smatspec^TM^ plus, Schiltigheim, France). In preliminary experiments, we tested RPMI-1640 culture and Sabouraud media. Similar data were obtained but the experiments were more reproducible with Sabouraud than with RPMI1640. Therefore, Sabouraud medium was used in the present study.

The yeasts (OD_600nm_ = 0.001) were then plated on 96-well microplates and treated with different concentrations of the peptides of interest. After 24 h incubation at 37 °C under agitation, yeast growth was assessed by optical density (OD_600nm_) using a microplate spectrophotometer (Multiscan EX, Thermo Fisher Scientific, Waltham, MA, USA). All tests were performed in triplicate. The MIC (minimal inhibitory concentration), defined as the lowest concentration of drug able to inhibit 100% of the growth of a pathogen, was determined for the different peptides against all previously described *Candida* strains, using a modified Gompertz model [40].

The same method was used to evaluate the MICs of peptide/bovine serum albumin combinations (BSA).

### 4.3. Cells Viability

Erythrocytes cells viability was assessed according to the previously reported method [17]. Cell viability was reported as the percentage of cell survival compared to positive controls. Each assay was tested in triplicate and data were compared to the positive control (*** *p* ≤ 0.001) with One way ANOVA.

### 4.4. Samples Preparation for Transmission Electron Microscopy

Precultures of sensitive and resistant *Candida albicans* strains were incubated for 2 h at 37 °C without shaking on glass bottom 24-well microplates previously treated with poly-*L*-lysine. The *L*-bCtl or *D*-bCtl peptides at concentrations of 1X MIC and 10X MIC were then added to the culture and incubated for 1 h at 37 °C with minimal shaking. The control samples correspond to the incubation of the different *Candida albicans* strains without any prior treatment. After 1 h incubation with or without each peptide of interest and a brief rinsing in 0.125 M cacodylate buffer, cells were fixed for 2 h 30 min in a 4% (*v*/*v*) glutaraldehyde buffered in 0.1 M sodium phosphate at pH 7.2. This step was followed with three 10 min washes in 0.1 M phosphate buffer at pH 7.2. Then the samples were post-fixed in a freshly prepared 2% (*v*/*v*) aqueous solution of potassium permanganate for 45 min at room temperature. The samples were washed three more times and dehydrated in graded series of ethanol of 7 min each. The composition of Spurr resin (Sigma Aldrich, Saint Quentin Fallavier, France) used was as follows: NSA (Nonenyl Succinic anhydride; 5.9 g), ERL4221 (cycloaliphatic epoxy-resin; 4.10 g), DER736 (Diglycidyl ether of polypropyleneglycol; 1.58 g), and DMAE (Dimethylaminoethanol; 0.1 g) as an accelerator. The samples were transferred successively in 1 vol Spurr resin/2 vol absolute ethanol for 15 min, then in 1 vol Spurr resin/1 vol absolute ethanol for 30 min and 2 times 1 h in 100% Spurr resin. Finally, after a new change overnight in 100% epoxy resin, the samples were successively placed in fresh resin at room temperature, 37 °C, 45 °C for 24 h and let 48 h in a 65 °C oven for polymerization.

### 4.5. Transmission Electron Microscopy

Ultrathin sections were performed using a Reichert Jung Ultra-cut E ultra-microtome (Leica Microsystems, Nanterre, France) equipped with a diamond knife. Ultrathin sections were collected on 100 mesh Formvar coated grids and stained with 5% uranyl acetate solution (Euromedex, Souffelweyersheim, France). After rinsing, the grids were stained with a 4% lead citrate solution for 10 min (Euromedex, Souffelweyersheim, France). Samples were observed with a Philips EM 208 instrument (FEI Company, Eindhoven, Netherlands) operating with an accelerating voltage of 70 KV. Images were recorded on Kodak SO163 film (Sigma Aldrich, Saint-Quentin Fallavier, France). After development, negatives were scanned at 600 dpi with EPSON/PERFECTION V750 PRO (Epson France, Lavallois Perret, France).

### 4.6. Quartz Crystal Microbalance Analysis

The construction of the multilayer film was monitored in situ with a quartz crystal microbalance (QCM) (Q-Sense E4, Goteborg, Sweden). A quartz crystal coated with a silica layer (SiO_2_) was used. After crystal cleaning with a 2% (*v*/*v*) Hellmanex solution (Hellma Analytics, Müllheim, Germany) for half an hour, combining with a rinsing step with milliQ water, 400 µL of PBS solution (pH 7.4) was injected into the measurement cell. After baseline stabilization, 400 μL of bovine serum albumin (BSA) in PBS (2 mg/mL at 37 °C) was deposited as the first layer and the deposition step was performed for 3 min. The stability of the first layer was assessed by performing a wash step for 5 min with PBS solution. The ability of bCts and bCtl (*L*-isomers) to interact with BSA was performed with a 3-min deposition step of 400 µL for each peptide at a concentration of 1 mg/mL, followed by a 5-min rinse step with PBS. The quartz crystal was excited at its fundamental frequency (about 5 MHz), as well as at the third, fifth, seventh, and ninth overtones (denoted by ν = 3, 5, 7, and 9 and corresponding to 15, 25, 35, and 45 MHz, respectively). Changes in the resonance frequencies (−Δfν) were measured at these four frequencies. An increase in the normalized frequency −Δfν/ν is often associated with a proportional increase in the mass coupled to the quartz crystal. The mass and molecular ratio of BSA/*L*-bCts or BSA/*L*-bCtl (*L*-isomers) were calculated according to Sauerbrey approximation [41].

### 4.7. Statistical Analysis

The proportions of lysed cells of each group tested were compared to the control experiment using the two-sided Fisher exact test and one-way analysis of variance test (ANOVA) with statistical significance set at *α* = 0.05.

### 4.8. Data Availability

Sequence of Catestatin and Cateslytin: Uni protKB, CMGA_Bovin P05059; CMGA_Human P10645.

## 5. Conclusions

Within the limitations of this original study, we can conclude that in vitro *D*-bCtl and the combination of BSA with *L*-bCts are potential candidates for the development of new antifungal drugs for the treatment of oral candidosis due to *Candida* and non-*Candida albicans*, without no detrimental side effects.

## Figures and Tables

**Table 1:** Susceptibility of *Candida* strains tested. EUCAST (European Committee on Antimicrobial Susceptibility Testing) breakpoints were used for Amphotericin B, Fluconazole, Voriconazole, and CLSI (Clinical and Laboratory Standards Institute) breakpoints for Caspofungin. “S”, “R”, and “I” correspond to susceptible standard dosing regimen, resistant, therapeutic failure with increased exposure and susceptible increased exposure, respectively.

Strain	Amphotericin B	Fluconazole	Voriconazole	Caspofungin
	MIC (µg/mL)	MIC (µg/mL)	MIC (µg/mL)	MIC (µg/mL)
***C. albicans*** **“S”**	0.19 **“S”**	0.125 **“S”**	0.002 **“S”**	0.094 **“S”**
***C. albicans*** **“R”**	0.125 **“S”**	>256 **“R”**	>32 **“R”**	0.047 **“S”**
***C. tropicalis*** **“S”**	0.125 **“S”**	0.5 **“S”**	0.047 **“S”**	0.125 **“S”**
***C. tropicalis*** **“R”**	0.125 **“S”**	96 **“R”**	1.5 **“R”**	0.125 **“S”**
***C. glabrata*** **“S”**	0.125 **“S”**	3 **“I”**	0.032 **“S”**	0.25 **“I”**
***C. glabrata*** **“R”**	0.5 **“S”**	>256 **“R”**	8 **“R”**	0.25 **“I”**
***C. lusitaniae*** **“S”**	0.75 **“S”**	0.38 **“S”**	0.012 **“S”**	0.125 **“S”**

**Table 2:** Antimicrobial activity against *C. albicans* of different bovine and human Cts-derived peptides. (a) Sequences and MIC (µg/mL) of the different peptides derived from bCts and bCtl against sensitive “S” and resistant “R” *C. albicans*; (b) sequences and MIC of the different peptides derived from hCts and hCtl against sensitive “S” and resistant “R” *C. albicans. L,* levogyre *and D,* dextrogyre isomers. * Values obtained from Dartevelle et al. [28].

**(a)**				
	**Peptide**	**Sequence**	**MIC (µg/mL) *C. albicans***	
			**“S”**	**“R”**
**bCts**	bCgA344–364	R S M ***R*** L S F R A R G Y G F R G P G L Q L	30	50
**bCtl**	*L*-bCgA344–358	R S M R L S F R A R G Y G F R	7.9 *	9.6
**P1**	***D***-**bCgA344–358**	**R S M R L S F R A R G Y G F R**	5.5 *	**9.6**
**P2**	*L*-bCgA344–351	R S M R L S F R	50	100
**P3**	*L*-bCgA344–351	*Ac*-R S M R L S F R	>100	>100
**P4**	*L*-bCgA344–351	*Pa*-R S M R L S F R	>100	>100
**P5**	*L*-bCgA347–352	R L S F R A-*W*	>100	>100
**P6**	*D*-bCgA347–352	R L S F R A-*W*	>100	>100
**P7**	*L*-bCgA348–353	*Ac*-L S F R A R	>100	>100
**P8**	*L*-bCgA348–351	L S F R	>100	>100
**P9**	*D*-bCgA348–351	L S F R	>100	>100
**P10**	*L*-bCgA348–351	*Ac*-L S F R	>100	>100
**P11**	*L*-bCgA348–351	*Pa*-L S F R	>100	>100
**(b)**				
	**Peptide**	**Sequence**	**MIC (µg/mL) *C. albicans***	
			**“S”**	**“R”**
**hCts**	hCgA352–372	S S M K L S F R A R A Y G F R G P G P Q L	>240	>240
**hCtl**	*L*-hCgA352–366	S S M K L S F R A R A Y G F R	50	50
**P12**	*L*-hCgA352–366	*Ac*-S S M K L S F R A R G Y G F R	>100	>100
**P13**	*L*-hCgA352–366	*Pa*-S S M K L S F R A R G Y G F R	>100	>100
**P14**	*L*-hCgA352–359	S S M K L S F R	>100	>100
**P15**	*L*-hCgA352–359	*Ac*-S S M K L S F R	>100	>100
**P16**	*L*-hCgA352–359	*Pa*-S S M K L S F R	>100	>100
**P17**	*L*-hCgA355–360	*Ac*-K L S F R A	>100	>100
**P18**	*L*-hCgA354–359	M K L S F R	>100	>100
**P19**	*L*-hCgA354–359	*Pa*-M K L S F R	>100	>100

**Table 3:** Antifungal activity of *L*-bCts, *L*-hCts, *L*-bCtl, and *D*-bCtl against various strains of *Candida*. The percentage of growth inhibition of the indicated yeasts in the presence of different concentrations of *L*-bCts, *L*-hCts, *L*-bCtl, and *D*-bCtl was determined by broth microdilution assays. Each MIC, defined as the lowest concentration of a drug able to inhibit 100% of fungal growth, was determined using a modified Gompertz model. Experiments were performed in triplicate. “S”: sensitive, “R”: resistant. * Values obtained from Dartevelle et al. [28].

Candida Strain.	*Candida albicans* “S”	*Candida albicans* “R”	*Candida tropicalis* “S”	*Candida tropicalis* “R”	*Candida glabrata* “S”	*Candida glabrata* “R”
**MIC of *L*-bCts (µg/mL)**	30	50	50	20	>100	>100
**MIC of *L*-hCts (µg/mL)**	>240	>240	>240	>240	>240	>240
**MIC of *L*-bCtl (µg/mL)**	7.9 *	9.6	9.8	2.0	38.2	61.4
**MIC of *D*-bCtl (µg/mL)**	5.5 *	9.6	8.1	2.0	13.4	15.0

## Data Availability

Not applicable.
